# Healthcare quality evaluation of tertiary public hospitals in ethnic border regions under China’s performance assessment system—based on the entropy weight TOPSIS method and RSR fuzzy set

**DOI:** 10.3389/fpubh.2025.1668065

**Published:** 2025-10-01

**Authors:** Junjie Huang, Yanlong Wu, HuiPing Pan, Haitao Yuan, Xinwen Liu, Huiyu Wang, Pingping Zeng, Zhong Tang, Pinghua Zhu

**Affiliations:** 1 Guangxi International Zhuang Medical Hospital, Nanning, China; 2 The First Affiliated Hospital of Guangxi Medical University, Nanning, China; 3 Guangxi Zhuang Autonomous Region Medical Management Service Guidance Center, Nanning, China; 4 Liuzhou Municipal Liutie Central Hospital, Liuzhou, China; 5 School of Nursing, Guangxi Medical University, Nanning, China; 6 School of Humanities and Social Sciences, Guangxi Medical University, Nanning, China

**Keywords:** tertiary public hospitals, healthcare quality, performance assessment, fuzzy set, Guangxi

## Abstract

**Objective:**

Performance evaluation is critical for improving healthcare quality and service delivery. This study analyzes the healthcare quality of tertiary hospitals across various cities in Guangxi under China’s public hospital performance assessment policy to identify influencing factors and propose targeted improvement strategies for the national evaluation system.

**Methods:**

The healthcare quality of general hospitals in Guangxi from 2019 to 2022 was evaluated using a fuzzy set entropy-weighted TOPSIS and RSR method, followed by a comprehensive city-level ranking.

**Results:**

Entropy-weighted TOPSIS revealed the top three weighted indicators: (1) number of referred-out patients, (2) low-risk group mortality rate, and (3) proportion of reviewed prescriptions. The quality of H7 and H11 improved significantly, while H9 consistently ranked in the top 4. The RSR stratification classified H1, H2, H8, and H9 as high-performing, H6 and H12 as low-performing, and H4, H5, H6, H12, and H13 as persistently below average for four consecutive years. Using the fuzzy set method, H1, H9, H11, and H1 were ranked as the highest-performing cities from 2019 to 2022, respectively.

**Conclusion:**

There are minor discrepancies among the three methodologies, but the trends remain largely consistent. The fuzzy-combined approach provides more robust evaluations, which offers actionable insights for healthcare quality enhancement and management standardization. Consequently, hospitals should improve the quality of services and sustain the core competitiveness of public hospitals by implementing tiered healthcare systems and standardized prescription review protocols.

## 1 Background

Healthcare quality is the key to the performance evaluation of tertiary hospitals. Management standards must be raised to foster ongoing improvements in healthcare quality and public service. In recent years, China has issued a series of important documents, including the “Guidelines for Strengthening the Performance Assessment of Tertiary Public Hospitals,” “Strategies for Promoting the High-Quality Development of Public Hospitals,” and “Announcement for Initiating a Comprehensive Campaign to Improve Healthcare Quality (2023–2025),” all of which underscore the critical importance of healthcare quality in public hospitals. Performance evaluation is not only a vital instrument for improving healthcare quality but also a cornerstone of the high-quality development trajectory and the modern management framework of public hospitals. Since 2009, China has been progressively developing theories and policies about the performance evaluation of public hospitals (1). The year 2019 witnessed the comprehensive implementation phase of performance evaluations in public hospitals (2). Concurrently, China introduced the “National Performance Assessment Manual for Tertiary Public Hospitals,” which appraises these institutions from four key dimensions: patient satisfaction, sustainable development, operational efficiency, and healthcare quality. The release of this manual signifies the first establishment of a cohesive national hospital performance assessment system in China. Healthcare Quality and patient safety constitute the bedrock of healthcare service (3). The performance evaluation of tertiary public hospitals prioritizes healthcare quality as the principal metric for assessments from four dimensions: role definition, service delivery, rational medication, and quality safety. These assessments aim to improve the management levels of healthcare institutions, enhance service delivery capabilities, protect public health rights and interests, and promote the high-quality development of the medical and health sector (4). The western regions of China are marked by a significantly smaller pool of healthcare resources and comparatively suboptimal hospital care quality. Guangxi ranks last among the six provinces of south-central China in terms of hospital beds, physicians, and nurses per 1,000 permanent residents. It has only 61,300 licensed physicians and ranks second to last in the size of the physician workforce, while its 1,481 comprehensive ICU beds fall short of Henan (the top-ranked province) by 4,343 beds. Similarly, other western autonomous regions—Xinjiang, Ningxia, and Tibet—perform poorly in their respective regional ranking (5). Moreover, multilingual ethnic settings and cross-border patient flows present additional challenges to healthcare delivery in these border minority areas. Addressing the challenge of how to meet the growing public health demands with these limited resources, while simultaneously advancing healthcare reforms through internal management system innovation, strengthening operational management, and improving care quality and equity in resource allocation, has become a major issue in China’s health management field. Resolving these issues is not only crucial for health improvements but also holds substantial significance for ethnic unity, making it a focal point of joint research attention in both academic and political circles in China (6).

### 1.1 Regarding the relationship between the quality of healthcare and the burden of medical expenditure

Studies have consistently shown that patients move to higher quality regions when local care quality cannot meet demands. Thorsen et al. found that higher severity strongly predicts travel to advanced hospitals (7). Xu et al. observed that migrants gravitate toward cities rich in tertiary centers (8). The 2021 Statistical Bulletin of China’s Medical Security Development reported that 162.7 million of 1.53 billion insured inpatient episodes occurred outside the home prefecture of patients, accounting for 10.6% of cases and 23.3% of inpatient expenditure. Thus, cross regional care incurs substantially higher costs per admission than local care.

### 1.2 Regarding the relationship between performance evaluation and healthcare quality improvement

China’s 2019 “Guidelines on Strengthening Performance Assessment of Tertiary Public Hospitals” explicitly stipulate that the evaluation results serve as the key determinants for government funding allocation, medical insurance reimbursement, total performance-based compensation, and remuneration, appointment, and disciplinary measures of hospital executives, along with a close combination of hospital accreditation and merit-based awards. Duan et al. conducted an in-depth analysis of the impact of performance evaluation systems on quality management. This revealed post-implementation improvements, including a 6.48% increase in Tier III and IV surgical procedures, 26.67 and 13.64% reductions in elective surgery complication rates and Class I incision infection rates, respectively, which demonstrated significant enhancements in operational efficiency and service quality (9). Wang Y further observed substantial policy effects, with a significant reduction in the intensity of antibiotic usage, a 20% reduction in outpatient waiting time, and a measurable improvement in patient satisfaction scores after the reform (10).

### 1.3 Regarding the application of healthcare quality assessment methodologies

The TOPSIS (Technique for Order Preference by Similarity to Ideal Solution) was first proposed by C. L. Hwang and K. Yoon in their seminal work Multiple Attribute Decision Making: Methods and Applications (1981). This method ranks alternatives by calculating their relative proximity to both ideal and negative-ideal solutions. Originally applied in marketing and business management, it has been subsequently adopted for health system evaluations. Notable applications include Wu XL and Liang Mingbin’s development of quality assessment criteria for cancer pain clinic records using TOPSIS-based weighting (11), and Beata Gavurova’s comparative analysis of health well-being across EU nations (12). The Rank Sum Ratio (RSR) method, introduced by Chinese statistician Professor Tian F in 1988, integrates nonparametric statistics with comprehensive evaluation (13). Its core mechanism involves rank transformation of raw data followed by RSR computation, enabling multidimensional assessment and ranking. This approach has gained particular prominence in healthcare quality evaluation (14). Representative applications include Wu et al.’s comparative evaluation of child healthcare services in China (15) and Liu’s maternal health service assessments using national health statistical yearbook data (16).

This study is the first to evaluate the healthcare quality levels of hospitals in western China by integrating the performance appraisal indicators of national tertiary public hospitals. Specifically, the contribution of this study is as follows: First, although existing studies have applied TOPSIS or RSR models to evaluate public hospitals, most have focused more on individual departments or hospitals. Different from other papers, this study assesses the healthcare quality levels of tertiary public hospitals in Guangxi from a city-to-city ranking perspective. This approach helps various cities to pinpoint weak links in healthcare quality management and prioritize areas for improvement. It also provides a scientific basis for local health administrative departments to analyze the current state of healthcare quality in tertiary general hospitals and to formulate national examination quality improvement plans. Second, in terms of model selection, TOPSIS and RSR models are integrated with the fuzzy set theory in a fuzzy union to derive a comprehensive ranking. Hence, the limitations of using a single evaluation method can be mitigated effectively.

## 2 Materials and methods

### 2.1 Data source

The data of this study are obtained from statistical and financial reports of 58 tertiary public hospitals in Guangxi from 2019 to 2022. Among them, there are 17 hospitals at the regional level, 36 at the municipal level, and 5 at the county level. Hospitals from different cities are categorized and integrated to encode 14 cities in the order of H1-H14.

### 2.2 Methods

#### 2.2.1 Evaluation indicators

The healthcare quality of tertiary public hospitals in Guangxi is evaluated according to the “National Tertiary Public Hospital Performance Assessment Manual (2024 Edition)” and relevant literature findings, which focus on four dimensions: service process, rational medication, quality safety, and functional positioning. Functional positioning requires tertiary hospitals to implement tiered diagnosis and treatment systems and to play a core role in regional healthcare, which reflects the hospital’s ability to treat critically ill patients and perform complex and highly challenging surgeries (17, 18). Quality safety, as the cornerstone of healthcare quality, is directly related to the health and well-being of the public. Rational medication, as a key aspect of healthcare quality supervision, fully embodies the functional positioning and public welfare nature of public hospitals (19). The service process is mainly assessed based on patient experience and the hospital’s level of informatization to evaluate the effectiveness of service improvement.

Following the principles of systematicity, scientificity, dynamism, availability, and policy orientation, this study ultimately constructs a healthcare quality evaluation index system for tertiary public general hospitals in Guangxi, including 18 secondary indicators (20–24), and assigns values to X1, X2, and X18 accordingly. The indicators X1-X6 reflect the hospital’s functional positioning and evaluate its role in the tiered healthcare delivery system. X7-X10 demonstrate clinical performance, X11-X16 effectively assess pharmaceutical management practices, while X17 and X18 measure patient service efficiency (Table 1).

**Table 1:** Healthcare quality evaluation indicator system for tertiary public general hospitals in Guangxi.

Primary indicators	Secondary indicators	Explanation of Indicators	Indicator attributes
Functional positioning	X1: Ratio of outpatient discharged patients	Ratio of outpatient discharged patients during the statistical period, reflecting the balance between outpatient and inpatient service volumes. The ratio of outpatient discharged patients = Number of outpatient visits ÷ Number of discharged patients during the same period.	Negative Indicator
	X2: Number of referred-out patients	During the assessment year, the number of patients transferred from tertiary public hospitals to secondary or primary care institutions, including both outpatients and inpatients. Number of referred-out patientss = Outpatient and Emergency referred-out patients + Inpatient referred-out patients	Positive Indicator
	X3: Proportion of Day Surgeries among Elective Surgeries	Proportion of day surgeries—defined as admission, operation, and discharge within 24 h—among total elective surgeries during the same period, reflecting healthcare efficiency. Proportion of day surgeries among elective surgeries = (Number of day surgeries ÷ Total number of elective surgeries among discharged patients during the same period) × 100%	Positive Indicator
	X4: Proportion of Surgical Discharges▲	Proportion of discharged patients who underwent surgical procedures. Surgical Discharge Ratio = (Number of surgical procedures performed on discharged patients ÷ Total number of discharged patients during the same period) × 100%	Positive Indicator
	X5: Proportion of Minimally Invasive Surgeries among Discharged Patients▲	Proportion of discharged patients undergoing minimally invasive procedures (e.g., laparoscopic, interventional), reflecting technological advancement. Proportion of Minimally Invasive Surgeries Among Discharged Patients = (Number of Minimally Invasive Surgical Procedures Performed on Discharged Patients ÷ Total Number of Surgical Procedures Performed on Discharged Patients During the Same Period) × 100%	Positive Indicator
	X6: Proportion of Grade IV Surgeries Among Discharged Patients▲	Proportion of Grade IV surgeries among discharged patients, indicating the hospital’s capacity for complex procedures. Proportion of Grade IV Surgeries Among Discharged Patients = (Number of Grade IV surgical procedures ÷ Total number of surgical procedures during the same period) × 100%	Positive Indicator
Quality and safety	X7: Surgical Complication Rate▲	Proportion of surgical patients developing complications (e.g., infection, hemorrhage), reflecting surgical safety. Surgical Complication Rate = (Number of surgical patients with complications ÷ Total number of surgical patients discharged during the same period) × 100%	Negative Indicator
	X8: Surgical Site Infection (SSI) Rate for Class I Incisions▲	Proportion of surgical site infections following Class I incisions (aseptic procedures). Surgical Site Infection (SSI) Rate for Class I Incisions = (Number of SSI cases in Class I wound surgeries ÷ Total number of Class I wound surgical procedures during the same period) × 100%	Negative Indicator
	X9: Interlaboratory Quality Assessment Pass Rate	Proportion of test items meeting proficiency criteria in interlaboratory quality assessment programs, reflecting laboratory accuracy. Interlaboratory Quality Assessment Pass Rate = (Number of test items meeting proficiency criteria in EQA programs organized by the National Center for Clinical Laboratories ÷ Total number of test items participating in EQA programs organized by the National Center for Clinical Laboratories during the same period) × 100%	Positive Indicator
	X10: Low-Risk Group Mortality Rate▲	Mortality rate among low-risk diagnostic groups (e.g., simple pneumonia, normal delivery), serving as an early warning for potential medical quality issues. Low-Risk Group Mortality Rate = (Number of deaths in low-risk group patients ÷ Total number of low-risk group patients) × 100%	Negative Indicator
Rational medication use	X11: Proportion of Reviewed Prescriptions to Total Prescriptions	Proportion of reviewed prescriptions to total prescriptions, reflecting the intensity of rational drug-use oversight. Proportion of Reviewed Prescriptions to Total Prescriptions = (Number of reviewed prescriptions ÷ Total number of prescriptions) × 100%	Positive Indicator
	X12: Antimicrobial Use Density▲	Antimicrobial Use Density (DDDs) of antimicrobials per 100 patient-days, indicating antimicrobial use density. Antimicrobial Use Density = (Total antimicrobial DDDs for inpatients ÷ Total patient-days during the same period) × 100	Negative Indicator
	X13: Proportion of Outpatient Prescriptions Containing Essential Medicines	Proportion of outpatient prescriptions containing essential medicines to total prescriptions, promoting the preferential use of essential drugs. Proportion of Outpatient Prescriptions Containing Essential Medicines = (Number of outpatient visits where essential medicines were prescribed ÷ Total number of outpatient visits during the same period) × 100%	Positive Indicator
	X14: Utilization Rate of Essential Medicines for Inpatients	Proportion of inpatients prescribed essential medicines during hospitalization. Utilization Rate of Essential Medicines for Inpatients = (Total number of discharged patients prescribed essential medicines ÷ Total number of discharged patients during the same period) × 100%	Positive Indicator
	X15: Proportion of Essential Medicines in Total Procurement Varieties	Proportion of essential medicine varieties in total drug procurement, reflecting essential drug availability. Proportion of Essential Medicines in Total Procurement Varieties = (Number of essential medicine varieties procured by the hospital ÷ Total number of drug varieties procured by the hospital during the same period) × 100%	Positive Indicator
	X16: Utilization Rate of Nationally Centralized Procurement Winning Drugs	Proportion of centrally procured winning drugs in total use of the same therapeutic category, reflecting policy implementation. Utilization Rate of Nationally Centralized Procurement Winning Drugs = (Volume of winning drugs used ÷ Total volume of same drug category used) × 100%	Positive Indicator
Service Process	X17: Outpatient Average Appointment Rate	Proportion of scheduled outpatient visits via any modality (online, telephone, referral, post-discharge follow-up, etc.) to total outpatient visits during the same period. Outpatient Average Appointment Rate = (Number of scheduled outpatient visits ÷ Total number of outpatient visits) × 100%	Positive Indicator
	X18: Electronic Medical Record (EMR) Application Functionality Tier Classification▲	Electronic Medical Record (EMR) Application Functionality Tier Classification assessed against National Health Commission standards, reflecting the level of informatization. Evaluation of Electronic Medical Record (EMR) and Clinical System Implementation Levels is conducted across three dimensions: system functionality realization, effective application scope, and data quality.	Positive Indicator

#### 2.2.2 Selection of indicator-weighting methods

Scholars have adopted various methodologies, such as the analytic hierarchy process and the coefficient of variation method, to determine the weights of indicators. Through a comprehensive literature review, the fundamental principles and relative strengths/limitations of the relevant models were analyzed, and the findings are summarized in Table 2 (25–29).

**Table 2:** Comparison of methods for determining indicator weights.

Dimension	Entropy-weighting method(EWM)	Analytic hierarchy process(AHP)	Coefficient of variation method
Core idea	Weights are determined by the dispersion inherent in the indicator data; greater dispersion yields more information and thus higher weight.	Experts pairwise-compare indicators to build a judgment matrix, whose principal eigenvector yields weights, emphasizing subjective judgments of relative importance.	Weights are assigned according to each indicator’s inherent variability; greater variability yields higher weight.
Weight nature	Objective weighting method	Subjective weighting method	Objective weighting method
Data requirements	Requires quantitative data, which must first be standardized.	Requires no raw quantitative data; expert scores on relative indicator importance are needed instead.	Requires quantitative data, which must first be standardized.
Advantages	1. Strong objectivity, entirely data-driven, free from human interference. 2. Adequate information: accurately reflects the actual information contained in the indicator data. 3. Transparent computation: the calculation process is straightforward and clear.	1. Handles complex, unstructured decisions and incorporates qualitative factors. 2. Integrates expert knowledge and experience. 3. Accounts for inter dependencies among indicators.	1. Highly objective, fully data-driven. 2. Conceptually simple and easy to compute. 3. Directly captures the relative volatility of indicator data.
Disadvantages	1. Highly sensitive to data quality, minor fluctuations can cause sharp weight changes. 2. May overlook real-world importance by reflecting only data dispersion.	1. Strongly subjective; results hinge on expert judgment. 2. Labor-intensive matrix construction and consistency checks when many indicators are involved. 3. Scores can vary widely across experts.	1. May ignore real importance by focusing only on variability. 2. Sensitive to extremes. 3. Ignores indicator correlations.
Applicable scenarios	Scenarios with sufficient data and the need for objective assessment of weights (e.g., performance evaluation, environmental quality assessment).	Scenarios with complex indicator systems that require expert judgment (e.g., project decision-making, risk assessment).	Scenarios where data fluctuation is pronounced and weights need to be determined quickly (e.g., financial indicator analysis, market volatility assessment).

#### 2.2.3 Entropy-weighted TOPSIS method

The entropy-weighted TOPSIS method combines the TOPSIS method and the entropy-weighted method. The entropy-weighted method employs standardized processing and objective weight determination to avoid human interference (30). The TOPSIS method ranks alternatives according to their distance from the ideal solution (31, 32). Zhou et al. applied entropy weight method (EWM) and fuzzy logic to identify key pollution parameters in water quality assessment, where indicator weights determined remediation priorities (33). Oluah et al. utilized entropy-weighted TOPSIS to establish thermal conductivity as the primary performance-limiting factor for phase-change materials in Trombe wall applications (34). Similarly, Zhan et al. employed entropy-weighted TOPSIS to reveal significant spatial distribution imbalances among tertiary hospitals in China’s Xinjiang region (35). In this study, the entropy-weighted TOPSIS method was used via STATA 17.0 to comprehensively evaluate healthcare quality data across different cities from 2019 to 2022. Data organization and preliminary calculations were performed using Excel 2021. The computational steps are as follows:

① The accurate original matrix X = (X_ij_) _n × m_ (where i = 1,2,…,n; j = 1,2,…,m), where X_ij_ is the value of the j-th indicator for the i-th object (36), m is the number of indicators, and n is the number of objects. For the five negative indicators: ratio of outpatient discharged patients, surgical complication rate, surgical site infection rate for class I incisions, low-risk group mortality rate, and antimicrobial use density, standardized values were obtained through range normalization. Specifically, Z_ij_^+^ represents the normalized score for positive indicators and Z_ij_^−^ for negative indicators, yielding the standardized matrix Z. The electronic medical record application functionality tier classification by Yang et al. (37) involves multiplying the level by 10 to better assess the service process in tertiary general hospitals.


$${Z_{\mathrm{ij}}}^{+}=\frac{x_{\mathrm{ij}}-x_{j\min}}{x_{j\max}-x_{j\min}}$$ (1)

$$\frac{{Z_{\mathrm{ij}}}^{-}=x_{j\max}-x_{\mathrm{ij}}}{x_{j\max}-x_{j\min}}$$ (2)

② Calculate the weight P_ij_, the entropy value e_j_, the difference coefficient d_j_, and the weight w_j_.


$$P_{\mathrm{ij}}=\frac{Z_{\mathrm{ij}}}{\sum_{i=1}^{n}Z_{\mathrm{ij}}}$$ (3)

$$e_{j}=-\frac{1}{1nn}\sum_{i=1}^{n}P_{\mathrm{ij}}1n\;P_{\mathrm{ij}},(j=1,2\ldots ,m)$$ (4)

$$d_{j}=1-e_{j}$$ (5)

$$W_{j}=\frac{d_{j}}{\sum_{j=1}^{m}d_{j}}$$ (6)

③ Construct the weighted TOPSIS matrix by multiplying each column of the normalized matrix Z with its corresponding weight; calculate the positive ideal solution Z^+^ and the negative ideal solution Z^−^; compute the Euclidean distance from each object to the positive ideal solution D_i_^+^ and to the negative ideal solution D_i_^−^; compute the relative closeness C_i_, with 0 ≤ C_i_ ≤ 1, where a higher C_i_ value is better.


$$Z^{+}=({Z_{1}}^{+},{Z_{2}}^{+},\cdots ,{Z_{m}}^{+})$$ (7)

$$Z^{-}=({Z_{1}}^{-},{Z_{2}}^{-},\cdots ,{Z_{m}}^{-})$$ (8)

$${D_{i}}^{+}=\sqrt{\sum_{j=1}^{m}{\left({Z_{j}}^{+}-Z_{\mathrm{ij}}\right)}^{2}}$$ (9)

$${D_{i}}^{-}=\sqrt{\sum_{j=1}^{m}{\left({Z_{j}}^{-}-Z_{\mathrm{ij}}\right)}^{2}}$$ (10)

$$C_{i}=\frac{{D_{i}}^{-}}{{D_{i}}^{+}+{D_{i}}^{-}}$$ (11)

#### 2.2.4 RSR model

The RSR (Rank-Sum Ratio) method is extensively applied in the medical and health field (38–40) for ranking and grading through rank transformation to obtain dimensionless statistical quantities. In this study, the comprehensive development level of healthcare quality C_i_ from 2019 to 2022 in each city is used instead of the RSR value. The RSR method is used to rank and grade the subjects using Excel 2021 and STATA 17 software. The computational steps are as follows:

① Prepare an n × m original matrix, where n represents the number of objects and m represents the number of indicators. Rank the indicators from smallest to largest according to the RSR method to calculate the rank matrix R, where a higher rank indicates better performance.

② Calculate the RSR value.


$$\mathrm{RSR}=\frac{1}{\mathrm{mn}}\sum_{j=1}^{m}\mathrm{Rij}$$ (12)

③ Construct the RSR frequency distribution. List the frequency f for each group, compute the cumulative frequency ∑f for each group, calculate the rank range and average rank $\bar{R}$ for RSR, compute the cumulative percentage $\bar{R}/n\times 100\%$ (with the last item recorded as $1-\frac{1}{4n}$ for correction), and calculate the probit value (the cumulative frequency plus 5 to obtain the standard normal deviation u).

④ Calculate the linear regression equation. The RSR value represents the dependent variable. The probit value represents the independent variable, which corresponds to the cumulative frequency in probability units. The linear regression equation is calculated through the least square method, that is:


RSR=a+b×Probit (13)

⑤ Conduct the regression equation test and obtain the estimated RSR values by running the regression equation, followed by grading and ranking of these values.

#### 2.2.5 Fuzzy set of entropy-weighted TOPSIS and RSR methods

To ensure the scientific nature of this study, the fuzzy set theory (41) is employed to perform a fuzzy union using the entropy-weighted TOPSIS and RSR methods. Generally, the weight ratio (W1: W2) is taken as 1:0, 0:1, 0.1:0.9, 0.5:0.5 and 0.9:0.1 (42), which indicates that the W1C_i_ × W2RSR value is calculated and ranked, where W1C_i_ × W2RSR ∈ (0, 1). The values closer to 1 indicate better results (43).

## 3 Results

### 3.1 Evaluation results using the entropy-weighted TOPSIS method

After standardizing indicators and performing normalized calculations, Table 3 displays the weighting of healthcare quality evaluation indicators. The four dimensions of healthcare quality indicators are weighted as follows: Functional Positioning (40.18%) > Rational Medication (31.42%) > Quality and Safety (21.592%) > Service Process (6.806%). Among the evaluation indicators, the top three are X2: number of referred-out patients (15.742%), X10: low-risk group mortality rate (11.158%), and X11: proportion of reviewed to total prescriptions (8.737%). The X2 serves as a core indicator for evaluating the implementation effectiveness of the tiered diagnosis and treatment policy, effectively highlighting hospitals’ functional positioning. The X10 represents a critical metric for healthcare safety that directly reflects institutional care quality. Meanwhile. The X11 establishes a robust medication safety safeguard by compelling hospitals to optimize the closed-loop process of “prescription review-evaluation-feedback-re-review.” The smallest weight is X1: Ratio of outpatient discharged patients (2.318%), as detailed in Table 3.

**Table 3:** Healthcare quality evaluation indicator system and weight calculation results.

Primary indicators	Secondary indicators	Information entropy value (e)	Information utility value (d)	Weight (%)
Functional positioning	X1: Ratio of outpatient discharged patients	0.966	0.034	2.318
	X2: Number of referred-out patients	0.766	0.234	15.742
	X3: Proportion of Day Surgeries among Elective Surgeries	0.884	0.116	7.784
	X4: Proportion of Surgical Discharges▲	0.946	0.054	3.651
	X5: Proportion of Minimally Invasive Surgeries among Discharged Patients▲	0.938	0.062	4.162
	X6: Proportion of Grade IV Surgeries Among Discharged Patients▲	0.903	0.097	6.523
Quality and safety	X7: Surgical Complication Rate▲	0.953	0.047	3.191
	X8: Surgical Site Infection (SSI) Rate for Class I Incisions▲	0.951	0.049	3.292
	X9: Interlaboratory Quality Assessment Pass Rate	0.941	0.059	3.951
	X10: Low-Risk Group Mortality Rate▲	0.834	0.166	11.158
Rational medication use	X11: Proportion of Reviewed Prescriptions to Total Prescriptions	0.87	0.13	8.737
	X12: Antimicrobial Use Density▲	0.958	0.042	2.86
	X13: Proportion of Outpatient Prescriptions Containing Essential Medicines	0.951	0.049	3.307
	X14: Utilization Rate of Essential Medicines for Inpatients	0.938	0.062	4.202
	X15: Proportion of Essential Medicines in Total Procurement Varieties	0.937	0.063	4.258
	X16: Utilization Rate of Nationally Centralized Procurement Winning Drugs	0.88	0.12	8.056
Service process	X17: Outpatient Average Appointment Rate	0.942	0.058	3.907
	X18: Electronic Medical Record (EMR) Application Functionality Tier Classification▲	0.957	0.043	2.899

“▲” indicates nationally monitored indicators.

The evaluation results indicate significant fluctuations in the C_i_ ranking among various cities over the observed period. Overall, the average C_i_ values from 2019 to 2022 were 0.48, 0.478, 0.433, and 0.411, respectively. Regions that failed to reach the average for four consecutive years included H4, H5, H6, H12, and H13, which suggested that the levels of healthcare quality in these areas require improvement. From a micro perspective, H1 returned to the forefront in 2022 after 2 years of fluctuating decline. H9 consistently ranked in the top 4 over the 4 years. H6 has been ranked at the bottom since 2019. The healthcare quality of H7 and H11 improved significantly, from 11th and 13th in 2019 to 5th and 7th in 2022, respectively. The healthcare quality of H10 and H12 declined significantly, each regressing by 8 places compared to 2019, as detailed in Table 4.

**Table 4:** Evaluation results of the relative closeness ci to healthcare quality in various cities of Guangxi from 2019 to 2022.

City	2019		2020		2021		2022	
	*C_i_*	Rank	*C_i_*	Rank	*C_i_*	Rank	*C_i_*	Rank
H1	0.560	1	0.527	4	0.457	6	0.573	1
H2	0.545	3	0.524	5	0.442	9	0.480	6
H3	0.506	5	0.528	2	0.444	8	0.553	3
H4	0.442	12	0.456	9	0.376	13	0.476	8
H5	0.461	10	0.447	10	0.417	10	0.471	9
H6	0.467	8	0.358	14	0.259	14	0.410	13
H7	0.445	11	0.516	6	0.479	2	0.518	5
H8	0.469	7	0.528	3	0.468	4	0.570	2
H9	0.557	2	0.562	1	0.469	3	0.532	4
H10	0.529	4	0.461	8	0.460	5	0.411	12
H11	0.413	13	0.481	7	0.550	1	0.479	7
H12	0.477	6	0.423	13	0.387	12	0.381	14
H13	0.467	9	0.436	12	0.399	11	0.467	10
H14	0.385	14	0.445	11	0.449	7	0.414	11

### 3.2 RSR evaluation results

The departments were ranked by order to display the standings of various regions under the healthcare quality evaluation indicators. The average RSR values of Guangxi from 2019 to 2022 were 0.508, 0.509, 0.451, and 0.511, respectively. The regions that failed to reach the average for four consecutive years were H4, H5, H6, H12, and H13, which is consistent with the results of the TOPSIS method. In terms of healthcare quality, the city that ranked first in both 2019 and 2020 was H9, while those that ranked first in 2021 and 2022 were H11 and H1, respectively. The most significant improvement in healthcare quality was seen in H7, which elevated 7 places in the ranking from 2019 to 2022 and now stands at 5th in Guangxi. See Table 5 for details.

**Table 5:** RSR, probit values, and RSR critical values for various cities from 2019 to 2022.

City	2019			2020			2021			2022		
	RSR (Rank)	Probit	RSR critical value	RSR (Rank)	Probit	RSR critical value	RSR (Rank)	Probit	RSR critical value	RSR (Rank)	Probit	RSR critical value
H1	0.607 (2)	6.465	0.598	0.572 (3)	6.068	0.569	0.487 (5)	5.566	0.482	0.625 (1)	7.100	0.662
H2	0.597 (3)	7.100	0.571	0.570 (4)	5.792	0.551	0.456 (9)	4.820	0.426	0.511 (6)	5.366	0.528
H3	0.543 (5)	5.180	0.536	0.568 (5)	5.566	0.536	0.463 (8)	5.000	0.440	0.604 (3)	6.068	0.582
H4	0.465 (11)	6.068	0.459	0.480 (9)	4.820	0.487	0.391 (12)	4.208	0.380	0.506 (8)	5.000	5.000
H5	0.486 (9)	5.366	0.485	0.469 (10)	4.634	0.475	0.435 (10)	4.634	0.412	0.505 (9)	4.820	0.486
H6	0.489 (8)	3.535	0.498	0.361 (14)	3.535	0.403	0.249 (14)	3.535	0.329	0.416 (13)	3.932	0.417
H7	0.464 (12)	5.000	0.444	0.557 (6)	5.366	0.523	0.505 (2)	6.465	0.550	0.561 (5)	5.180	0.543
H8	0.494 (7)	4.634	0.510	0.578 (2)	6.465	0.595	0.496 (4)	5.792	0.500	0.624 (2)	6.465	0.613
H9	0.608 (1)	5.792	0.641	0.608 (1)	7.100	0.637	0.497 (3)	6.068	0.520	0.573 (4)	5.792	0.561
H10	0.577 (4)	4.434	0.552	0.487 (8)	5.000	0.499	0.481 (6)	5.366	0.467	0.426 (12)	4.208	0.439
H11	0.415 (13)	4.208	0.425	0.510 (7)	5.180	0.511	0.607 (1)	7.100	0.598	0.508 (7)	5.180	0.514
H12	0.507 (6)	5.566	0.523	0.445 (13)	3.932	0.429	0.395 (11)	4.434	0.397	0.382 (14)	3.535	0.387
H13	0.483 (10)	3.932	0.473	0.463 (11)	4.434	0.462	0.387 (13)	3.932	0.359	0.488 (10)	4.634	0.471
H14	0.378 (14)	4.820	0.397	0.460 (12)	4.208	0.447	0.466 (7)	5.180	0.453	0.429 (11)	4.434	0.456

According to the optimal grading principle of the RSR method (44), the medical development levels of various cities from 2019 to 2022 were categorized into three grades: good, moderate, and poor. The grading results are shown in Table 6. In each year, there were 2 cities with poor levels, 9 cities with moderate levels, and 3 cities with good levels.

**Table 6:** RSR method grading results for various cities from 2019 to 2022.

Year	Grade	Percentile threshold	Probit	RSR critical value	Bin results
2019	Poor	<15.866	<4	<0.4293	H11, H14
	Moderate	15.866~	4~	0.4293 ~	H3, H4, H5, H6, H7, H8, H10, H12, H13
	Good	84.134~	6~	0.5662 ~	H1, H2, H9
2020	Poor	<15.866	<4	<0.4337	H6, H12
	Moderate	15.866~	4~	0.4337 ~	H2, H3, H4, H5, H7, H10, H11, H13, H14
	Good	84.134~	6~	0.5649 ~	H1, H8, H9
2021	Poor	<15.866	<4	<0.3641	H6, H13
	Moderate	15.866~	4~	0.3641 ~	H1, H2, H3, H4, H5, H8, H10, H12, H14
	Good	84.134~	6~	0.5153 ~	H7, H9, H11
2022	Poor	<15.866	<4	<0.4225	H6, H12
	Moderate	15.866~	4~	0.4225 ~	H2, H4, H5, H7, H9, H10, H11, H13, H14
	Good	84.134~	6~	0.5769 ~	H1, H3, H8

### 3.3 Fuzzy set evaluation results

**Figure 1:**
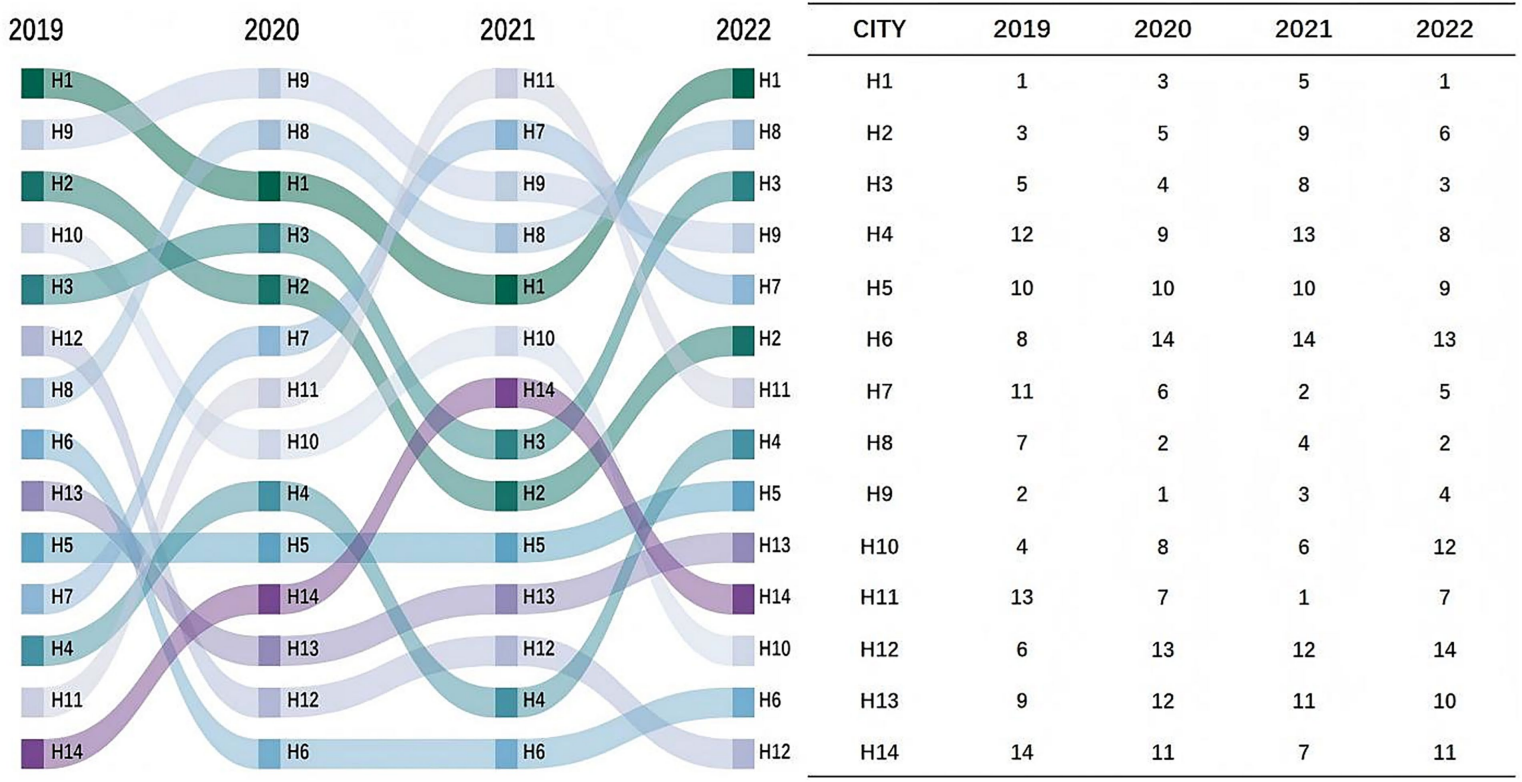
Healthcare quality ranking of cities in Guangxi from 2019 to 2022.

The fuzzy set theory (FST) was used to integrate the entropy-weighted TOPSIS and RSR methods to ensure the scientific validity of the results in this study. From 2019 to 2022, the healthcare quality of tertiary public hospitals in Guangxi was evaluated across five grading categories. The results of the C_i_ and RSR values will not be repeated here. Under the weight of 0.1C_i_ + 0.9RSR, the top-ranked city of both 2019 and 2020 was H9, and those of 2021 and 2022 were H11 and H1, respectively. The ranking results were consistent under the weights of 0.5C_i_ + 0.5RSR and 0.9C_i_ + 0.1RSR. The top-ranked cities over the 4 years were H1, H9, H11, and H1, respectively. Following the “more is better” principle, the comprehensive ranking of all cities was determined (45). The top-ranked cities from 2019 to 2022 were H1, H9, H11, and H1, respectively. For detailed results, see Table 7 and Figure 1.

**Table 7:** Evaluation results using fuzzy set method.

Year	City	C_i_ value	Rank	RSR value	Rank	0.1C_i_ + 0.9RSR	Rank	0.5C_i_ + 0.5RSR	Rank	0.9C_i_ + 0.1RSR	Rank	Comprehensive ranking
2019	H1	0.560	1	0.607	2	0.602	2	0.584	1	0.565	1	1
	H2	0.545	3	0.597	3	0.591	3	0.571	3	0.550	3	3
	H3	0.506	5	0.543	5	0.539	5	0.524	5	0.510	5	5
	H4	0.442	12	0.465	11	0.462	11	0.453	12	0.444	12	12
	H5	0.461	10	0.486	9	0.484	9	0.473	10	0.463	10	10
	H6	0.467	8	0.489	8	0.487	8	0.478	8	0.470	8	8
	H7	0.445	11	0.464	12	0.462	12	0.455	11	0.447	11	11
	H8	0.469	7	0.494	7	0.491	7	0.482	7	0.472	7	7
	H9	0.557	2	0.608	1	0.603	1	0.583	2	0.562	2	2
	H10	0.529	4	0.577	4	0.572	4	0.553	4	0.534	4	4
	H11	0.413	13	0.415	13	0.415	13	0.414	13	0.413	13	13
	H12	0.477	6	0.507	6	0.504	6	0.492	6	0.480	6	6
	H13	0.467	9	0.483	10	0.481	10	0.475	9	0.468	9	9
	H14	0.385	14	0.378	14	0.379	14	0.382	14	0.384	14	14
2020	H1	0.527	4	0.572	3	0.567	3	0.549	3	0.549	3	3
	H2	0.524	5	0.570	4	0.566	4	0.547	5	0.547	5	5
	H3	0.528	2	0.568	5	0.564	5	0.548	4	0.548	4	4
	H4	0.456	9	0.480	9	0.478	9	0.468	9	0.468	9	9
	H5	0.447	10	0.469	10	0.466	10	0.458	10	0.458	10	10
	H6	0.358	14	0.361	14	0.361	14	0.360	14	0.360	14	14
	H7	0.516	6	0.557	6	0.553	6	0.537	6	0.537	6	6
	H8	0.528	3	0.578	2	0.573	2	0.553	2	0.553	2	2
	H9	0.562	1	0.608	1	0.604	1	0.585	1	0.585	1	1
	H10	0.461	8	0.487	8	0.484	8	0.474	8	0.474	8	8
	H11	0.481	7	0.510	7	0.507	7	0.496	7	0.496	7	7
	H12	0.423	13	0.445	13	0.442	13	0.434	13	0.434	13	13
	H13	0.436	12	0.463	11	0.460	11	0.449	12	0.449	12	12
	H14	0.445	11	0.460	12	0.458	12	0.452	11	0.452	11	11
2021	H1	0.457	6	0.487	5	0.484	5	0.472	5	0.460	6	5
	H2	0.442	9	0.456	9	0.455	9	0.449	9	0.443	9	9
	H3	0.444	8	0.463	8	0.461	8	0.453	8	0.445	8	8
	H4	0.376	13	0.391	12	0.389	12	0.383	13	0.378	13	13
	H5	0.417	10	0.435	10	0.433	10	0.426	10	0.419	10	10
	H6	0.259	14	0.249	14	0.250	14	0.254	14	0.258	14	14
	H7	0.479	2	0.505	2	0.503	2	0.492	2	0.482	2	2
	H8	0.468	4	0.496	4	0.493	4	0.482	4	0.471	4	4
	H9	0.469	3	0.497	3	0.494	3	0.483	3	0.472	3	3
	H10	0.460	5	0.481	6	0.479	6	0.471	6	0.462	5	6
	H11	0.550	1	0.607	1	0.602	1	0.578	1	0.555	1	1
	H12	0.387	12	0.395	11	0.395	11	0.391	12	0.388	12	12
	H13	0.399	11	0.387	13	0.388	13	0.393	11	0.398	11	11
	H14	0.449	7	0.466	7	0.464	7	0.457	7	0.451	7	7
2022	H1	0.573	1	0.625	1	0.620	1	0.599	1	0.578	1	1
	H2	0.480	6	0.511	6	0.508	6	0.496	6	0.483	6	6
	H3	0.553	3	0.604	3	0.599	3	0.578	3	0.558	3	3
	H4	0.476	8	0.506	8	0.503	8	0.491	8	0.479	8	8
	H5	0.471	9	0.505	9	0.501	9	0.488	9	0.475	9	9
	H6	0.410	13	0.416	13	0.415	13	0.413	13	0.411	13	13
	H7	0.518	5	0.561	5	0.557	5	0.540	5	0.522	5	5
	H8	0.570	2	0.624	2	0.619	2	0.597	2	0.575	2	2
	H9	0.532	4	0.573	4	0.569	4	0.552	4	0.536	4	4
	H10	0.411	12	0.426	12	0.424	12	0.419	12	0.413	12	12
	H11	0.479	7	0.508	7	0.505	7	0.494	7	0.482	7	7
	H12	0.381	14	0.382	14	0.382	14	0.382	14	0.382	14	14
	H13	0.467	10	0.488	10	0.486	10	0.478	10	0.470	10	10
	H14	0.414	11	0.429	11	0.427	11	0.421	11	0.416	11	11

### 3.4 Validation

To demonstrate the differences in healthcare quality evaluation among the 14 cities, we performed an analysis of variance (ANOVA) on the evaluation scores derived from the TOPSIS, RSR, and Fuzzy methods. The results showed that the *p*-values for all five sets of evaluation scores were less than 0.01, indicating statistically significant differences in healthcare quality assessments. See Table 8 for details. To ensure robustness, the fuzzy-set method was subjected to a sensitivity test. The original weights were perturbed by replacing the combination of 0.1 Ci + 0.9RSR and 0.9 Ci + 0.1RSR with 0.2 Ci + 0.8RSR and 0.7 Ci + 0.3RSR, respectively, and 14 cities were re-ranked accordingly. The resulting rankings remained identical to those produced by the FUZZY STE model, which confirmed its reliability (Table 9).

**Table 8:** Analysis of differences in healthcare quality evaluation.

Characteristic factors	City	Mean±SD	F	LSD
Ci value	H1	0.53 ± 0.05	3.740**	H9 > H1 > H8 > H3 > H2 > H7 > H11 > H10 > H5 > H13 > H4 > H14 > H12 > H6
	H2	0.5 ± 0.05		
	H3	0.51 ± 0.05		
	H4	0.44 ± 0.04		
	H5	0.45 ± 0.02		
	H6	0.37 ± 0.09		
	H7	0.49 ± 0.03		
	H8	0.51 ± 0.05		
	H9	0.53 ± 0.04		
	H10	0.47 ± 0.05		
	H11	0.48 ± 0.06		
	H12	0.42 ± 0.04		
	H13	0.44 ± 0.03		
	H14	0.42 ± 0.03		
RSR value	H1	0.57 ± 0.06	3.730**	H1 > H9 > H8 > H3 > H2 > H7 > H11 > H10 > H5 > H4 > H13 > H14 > H12 > H6
	H2	0.53 ± 0.06		
	H3	0.54 ± 0.06		
	H4	0.46 ± 0.05		
	H5	0.47 ± 0.03		
	H6	0.38 ± 0.1		
	H7	0.52 ± 0.05		
	H8	0.55 ± 0.06		
	H9	0.57 ± 0.05		
	H10	0.49 ± 0.06		
	H11	0.51 ± 0.08		
	H12	0.43 ± 0.06		
	H13	0.46 ± 0.05		
	H14	0.43 ± 0.04		
0.1 Ci + 0.9RSR	H1	0.56 ± 0.06	3.736**	H1 > H9 > H8 > H3 > H2 > H7 > H11 > H10 > H5 > H4 > H13 > H14 > H12 > H6
	H2	0.53 ± 0.06		
	H3	0.54 ± 0.06		
	H4	0.46 ± 0.05		
	H5	0.47 ± 0.03		
	H6	0.38 ± 0.1		
	H7	0.52 ± 0.04		
	H8	0.54 ± 0.06		
	H9	0.56 ± 0.05		
	H10	0.49 ± 0.06		
	H11	0.5 ± 0.07		
	H12	0.43 ± 0.05		
	H13	0.45 ± 0.04		
	H14	0.43 ± 0.04		
0.5 Ci + 0.5RSR	H1	0.55 ± 0.06	3.749**	H1 > H9 > H8 > H3 > H2 > H7 > H11 > H10 > H5 > H13 > H4 > H14 > H12 > H6
	H2	0.52 ± 0.05		
	H3	0.53 ± 0.05		
	H4	0.45 ± 0.05		
	H5	0.46 ± 0.03		
	H6	0.38 ± 0.09		
	H7	0.51 ± 0.04		
	H8	0.53 ± 0.06		
	H9	0.55 ± 0.05		
	H10	0.48 ± 0.06		
	H11	0.5 ± 0.07		
	H12	0.42 ± 0.05		
	H13	0.45 ± 0.04		
	H14	0.43 ± 0.03		
0.9 Ci + 0.1RSR	H1	0.54 ± 0.05	3.743**	H9 > H1 > H8 > H3 > H2 > H7 > H11 > H10 > H5 > H13 > H4 > H14 > H12 > H6
	H2	0.51 ± 0.05		
	H3	0.52 ± 0.05		
	H4	0.44 ± 0.05		
	H5	0.46 ± 0.03		
	H6	0.38 ± 0.09		
	H7	0.5 ± 0.04		
	H8	0.52 ± 0.05		
	H9	0.54 ± 0.05		
	H10	0.47 ± 0.05		
	H11	0.49 ± 0.06		
	H12	0.42 ± 0.05		
	H13	0.45 ± 0.04		
	H14	0.43 ± 0.03		

**Table 9:** Robustness test of the fuzzy set method.

Year	City	C_i_ value	Rank	RSR value	Rank	0.2C_i_ + 0.8RSR	Rank	0.5C_i_ + 0.5RSR	Rank	0.7C_i_ + 0.3RSR	Rank	Robustness analysis ranking	Original comprehensive ranking
2019	H1	0.56	1	0.607	2	0.5976	2	0.584	1	0.5741	1	1	1
	H2	0.545	3	0.597	3	0.5866	3	0.571	3	0.5606	3	3	3
	H3	0.506	5	0.543	5	0.5356	5	0.524	5	0.5171	5	5	5
	H4	0.442	12	0.465	11	0.4604	11	0.453	12	0.4489	12	12	12
	H5	0.461	10	0.486	9	0.481	9	0.473	10	0.4685	10	10	10
	H6	0.467	8	0.489	8	0.4846	8	0.478	8	0.4736	8	8	8
	H7	0.445	11	0.464	12	0.4602	12	0.455	11	0.4507	11	11	11
	H8	0.469	7	0.494	7	0.489	7	0.482	7	0.4765	7	7	7
	H9	0.557	2	0.608	1	0.5978	1	0.583	2	0.5723	2	2	2
	H10	0.529	4	0.577	4	0.5674	4	0.553	4	0.5434	4	4	4
	H11	0.413	13	0.415	13	0.4146	13	0.414	13	0.4136	13	13	13
	H12	0.477	6	0.507	6	0.501	6	0.492	6	0.486	6	6	6
	H13	0.467	9	0.483	10	0.4798	10	0.475	9	0.4718	9	9	9
	H14	0.385	14	0.378	14	0.3794	14	0.382	14	0.3829	14	14	14
2020	H1	0.527	4	0.572	3	0.563	3	0.549	3	0.5405	3	3	3
	H2	0.524	5	0.57	4	0.5608	4	0.547	5	0.5378	5	5	5
	H3	0.528	2	0.568	5	0.56	5	0.548	4	0.54	4	4	4
	H4	0.456	9	0.48	9	0.4752	9	0.468	9	0.4632	9	9	9
	H5	0.447	10	0.469	10	0.4646	10	0.458	10	0.4536	10	10	10
	H6	0.358	14	0.361	14	0.3604	14	0.36	14	0.3589	14	14	14
	H7	0.516	6	0.557	6	0.5488	6	0.537	6	0.5283	6	6	6
	H8	0.528	3	0.578	2	0.568	2	0.553	2	0.543	2	2	2
	H9	0.562	1	0.608	1	0.5988	1	0.585	1	0.5758	1	1	1
	H10	0.461	8	0.487	8	0.4818	8	0.474	8	0.4688	8	8	8
	H11	0.481	7	0.51	7	0.5042	7	0.496	7	0.4897	7	7	7
	H12	0.423	13	0.445	13	0.4406	13	0.434	13	0.4296	13	13	13
	H13	0.436	12	0.463	11	0.4576	11	0.449	12	0.4441	12	12	12
	H14	0.445	11	0.46	12	0.457	12	0.452	11	0.4495	11	11	11
2021	H1	0.457	6	0.487	5	0.481	5	0.472	5	0.466	5	5	5
	H2	0.442	9	0.456	9	0.4532	9	0.449	9	0.4462	9	9	9
	H3	0.444	8	0.463	8	0.4592	8	0.453	8	0.4497	8	8	8
	H4	0.376	13	0.391	12	0.388	13	0.383	13	0.3805	13	13	13
	H5	0.417	10	0.435	10	0.4314	10	0.426	10	0.4224	10	10	10
	H6	0.259	14	0.249	14	0.251	14	0.254	14	0.256	14	14	14
	H7	0.479	2	0.505	2	0.4998	2	0.492	2	0.4868	2	2	2
	H8	0.468	4	0.496	4	0.4904	4	0.482	4	0.4764	4	4	4
	H9	0.469	3	0.497	3	0.4914	3	0.483	3	0.4774	3	3	3
	H10	0.46	5	0.481	6	0.4768	6	0.471	6	0.4663	6	6	6
	H11	0.55	1	0.607	1	0.5956	1	0.578	1	0.5671	1	1	1
	H12	0.387	12	0.395	11	0.3934	11	0.391	12	0.3894	12	12	12
	H13	0.399	11	0.387	13	0.3894	12	0.393	11	0.3954	11	11	11
	H14	0.449	7	0.466	7	0.4626	7	0.457	7	0.4541	7	7	7
2022	H1	0.573	1	0.625	1	0.6146	1	0.599	1	0.5886	1	1	1
	H2	0.48	6	0.511	6	0.5048	6	0.496	6	0.4893	6	6	6
	H3	0.553	3	0.604	3	0.5938	3	0.578	3	0.5683	3	3	3
	H4	0.476	8	0.506	8	0.5	8	0.491	8	0.485	8	8	8
	H5	0.471	9	0.505	9	0.4982	9	0.488	9	0.4812	9	9	9
	H6	0.41	13	0.416	13	0.4148	13	0.413	13	0.4118	13	13	13
	H7	0.518	5	0.561	5	0.5524	5	0.54	5	0.5309	5	5	5
	H8	0.57	2	0.624	2	0.6132	2	0.597	2	0.5862	2	2	2
	H9	0.532	4	0.573	4	0.5648	4	0.552	4	0.5443	4	4	4
	H10	0.411	12	0.426	12	0.423	12	0.419	12	0.4155	12	12	12
	H11	0.479	7	0.508	7	0.5022	7	0.494	7	0.4877	7	7	7
	H12	0.381	14	0.382	14	0.3818	14	0.382	14	0.3813	14	14	14
	H13	0.467	10	0.488	10	0.4838	10	0.478	10	0.4733	10	10	10
	H14	0.414	11	0.429	11	0.426	11	0.421	11	0.4185	11	11	11

## 4 Discussion

### 4.1 The rationality of using a fuzzy set of entropy-weighted TOPSIS and RSR

Entropy-weighted TOPSIS and the RSR method are widely used in health management. Relying on a single evaluation technique, however, inevitably introduces limitations that can distort the results (46). Entropy weighting objectively quantifies the relative importance of each indicator and avoids subjective bias (47). TOPSIS quantifies the distance between each alternative and the positive and negative ideal solutions, thereby capturing differences among schemes and enhancing reliability. Yet this approach is sensitive to extreme values and struggles to convey the differential impact of individual indicators (48). Chenghui et al. incorporated deliberately extreme values into real operational data from 24 tertiary hospitals and observed that TOPSIS misclassified three hospitals (49). The RSR method, which uses ranks rather than raw values, effectively dampens the influence of outliers and highlights minor fluctuations, but the ranking process discards metric information and weakens data utilization, this limitation also emerged in our study, as the stratification failed to reflect subtle shifts in the RSR values across cities (Table 6). To address the limitations of using the TOPSIS or RSR method alone, this study employs the fuzzy set theory to perform a fuzzy integration of the two methods. Based on the weight ratios of 1:0, 0:1, 0.1:0.9, 0.5:0.5, and 0.9:0.1, this study ranks various cities in Guangxi across different years. The final ranking is determined using a comprehensive evaluation approach based on the “majority principle.” This method not only takes full advantage of the raw data but also mitigates the impact of outliers, which enhances the reliability and accuracy of the evaluation results.

### 4.2 The issue of unbalanced healthcare quality levels across various cities in Guangxi becomes prominent

This study calculated the mean values across five weight ratios—1:0, 0:1, 0.1:0.9, 0.5:0.5, and 0.9:0.1—over 4 years and determined the proportion of hospitals that failed to meet the average values. In the TOPSIS evaluation model, the proportion of cities that failed to reach the mean values from 2019 to 2022 was 64.29, 50, 35.71, and 64.29%, respectively. In the RSR model, the proportion of cities below the average was 64.29, 50, 35.71, and 57.14%, respectively. The results calculated under the weight ratio of 0.1C_i_ + 0.9RSR were consistent with the RSR model. Under the weight ratio of 0.5C_i_ + 0.5RSR, the proportion of cities that failed to reach the average was 57.14, 50, 35.71, and 57.14%, respectively. The results under the weight ratio of 0.9C_i_ + 0.1RSR were consistent with the TOPSIS model. It is evident that, except for 2021, more than 50% of the cities did not meet the average standard in other years. In the evaluation model, the values of the top-ranked cities were more than double those of the lowest-ranked cities, which indicated a significant imbalance in the development of healthcare quality levels in Guangxi. H1, H2, H8, and H9 achieved better rankings, while H6 and H12 ranked relatively behind. The reasons included Guangxi’s relatively weak economic foundation, differences in population size, economic development levels of various regions, and health financial investment, which certainly led to disparities in healthcare quality levels. In 2021, there was a drastic change in the ranking of cities across Guangxi, since the region was in a critical phase of epidemic prevention and control. Additionally, labor expenses of medical staff, consumption of protective materials, tasks of nucleic acid testing, and responsibilities for epidemic prevention and control varied across hospitals at different levels. Areas with higher healthcare quality levels also undertook heavier tasks of epidemic prevention, and these external factors affected the development of local healthcare quality to varying degrees. H8, with only three tertiary hospitals, still achieved a better grade, which demonstrated that while focusing on external conditions, hospitals must also identify their own positioning and development direction (50). Cities H7 and H11 demonstrated consistent upward trends in healthcare quality indicator rankings during the four-year period, which prompted the targeted investigation. H7 implemented the “Implementation Plan for High-Quality Development of Public Hospitals,” requiring that all the municipal tertiary public hospitals improve the national performance evaluation ranking by at least 100 positions. Notably, H7 was one of the first cities in Guangxi to adopt the innovative “1 + N dynamic performance salary allocation mechanism.” These policy interventions collectively contributed to significant improvements in H7’s healthcare quality metrics. In-depth analysis of poorly-performing cities H6 and H12 revealed three common deficiencies. First, jurisdictions experienced severe underinvestment: H6’s healthcare project expenditure fell by 35% between 2020 and 2022, while H12’s health budget was only 73% of the Guangxi average. Second, the staff shortage was severe. In 2022, H6 had 443 unfilled posts, 80% of which were in imaging, anaesthesiology and critical care, while H12 lost 92 senior professionals between 2020 and 2022. This made the anaesthesiologist number below the national baseline for tertiary hospitals. Third, digital maturity lagged: none of H6 hospitals achieved Level 4 EMR capability, and H12’s tertiary hospitals had the lowest DRG upload completeness at 72%. Consequently, both municipal governments should increase fiscal commitments to health, accelerate information-system upgrades, and cooperate with universities to break disciplinary silos, strengthen professional capacity, and reduce patient outflow, while simultaneously securing a larger and more stable health workforce. Cities with poor performance in healthcare quality should fully leverage the targeted support of the State Ethnic Affairs Commission, utilize the strengths of their paired tertiary hospitals, and pursue cross-border cooperation with neighboring countries.

### 4.3 The indicators of “functional positioning” and “rational medication” affect healthcare quality

In the dimensions of healthcare quality assessment, the primary indicators with higher weights are functional positioning and rational medication, with weights of 40.18 and 31.42%, respectively. Among the secondary indicators, the top three are X2: number of referred-out patients (15.742%), X10: low-risk group mortality rate (11.158%), and X11: proportion of reviewed to total prescriptions (8.737%). X2 and X11 are the high-weight primary indicators, while X10 pertains to quality and safety. This suggests that when cities focus on high-weight primary indicator groups, they must also balance the advancement of other significant indicators. The survey samples consist exclusively of tertiary and above general hospitals. Functional positioning is primarily focused on the treatment of critically ill patients and complex medical conditions (51). Data from field research indicate that regions categorized as “good” have a concentration of superior medical resources and exert a significant gravitational pull on patients from surrounding cities. These areas have an adequate number of patients to adjust treatment structures, which allows hospitals to focus on complex regional diseases, enhance service provision, and improve indicators such as “proportion of surgical discharges,” “proportion of minimally invasive surgeries among discharged patients,” and “proportion of grade IV surgeries among discharged patients”. Regions with a “moderate” level possess a certain volume of healthcare resources that can cover common diseases and treatment needs for more challenging surgeries. These areas should ensure basic medical service provision while expanding the scope of medical service, which may optimize service structures and increase the supply of specialized medical services to meet patients’ diverse and personalized healthcare needs. Compared to the aforementioned regions, areas with a “poor” level still have a gap in healthcare service provision, with deficiencies in complex surgeries, rare disease treatment, and high-end medical services. It is recommended that these areas should strengthen the construction of key medical disciplines and specialties, create advantageous specialties, enhance the brand influence of hospitals, and focus on tackling prevalent and frequently referred diseases to improve core competitiveness. Additionally, hospitals should also respond to the construction of a medical consortium, implement a tiered diagnosis and treatment system, and facilitate the downward transfer of high-quality medical resources. With a focus on the treatment of complex and acute-phase diseases, common, frequent, and stable or recovery-phase patients can be gradually transferred downward through the “urgent and chronic disease separation, upward and downward linkage.” The aim is to enhance the efficiency of medical resource utilization and clarify the positioning of tertiary public hospitals, which thus enhances the development level of functional positioning. On the other hand, to promote the transformation of pharmaceutical service models, all tertiary hospitals should standardize prescription review management systems, regularly organize special groups to review prescriptions and medical orders, and involve physicians, pharmacists, nurses, and patients in the medication process. In 2022, the 25th percentile, median, and 75th antimicrobial use density across various cities in Guangxi were 36.005, 36.875, and 38.538, respectively. Regions with a “poor” level performed better in controlling antibiotic use, and H12 had the best value of 32.87 in the entire region. In contrast, regions with a “good” level had a value of 36.65 exceeding both the median and average values of Guangxi. It is recommended that these areas reasonably increase the proportion of basic medication use and standardize medication practices. Additionally, irrational prescriptions should be identified through a combination of big-data intelligent review and manual review. The intervention and follow-up management of over-prescribed medications is also required to ensure clinical medication safety (52).

## 5 Limitations

There are several limitations of this study. Firstly, the data obtained span a relatively short period only from 2019 to 2022. However, the results are still useful for assessing the development of healthcare quality levels across various cities. In the future, it is necessary to monitor data over additional years for further validation. Secondly, the use of the entropy-weighted TOPSIS and RSR methods for objective weighting of evaluation indicators may, to some extent, overlook the inherent importance of the raw data and the indicators themselves, which leads to potential biases. However, the fuzzy integration of the two methods can help mitigate such an impact to a certain degree. Thirdly, the evaluation is limited to tertiary public hospitals in Guangxi and then restricts the generalizability of the results. Although such limitations are common in research, hospitals from Guangxi are fortunately included in this study. Besides, Guangxi is representative of western China at the average levels of economic and social development. Hence, the results of this study are still applicable to the actual situations of various cities in western China.

## 6 Conclusion

Using the fuzzy set of entropy-weighted TOPSIS and RSR methods, this study evaluates the healthcare quality levels of 59 tertiary public hospitals in Guangxi from 2019 to 2022 and analyzes the ranking of healthcare quality across 14 prefecture-level cities. There are significant fluctuations in healthcare quality across different years but with a relatively stable healthcare quality. The evaluation results of healthcare quality in different regions using the entropy-weighted TOPSIS and RSR methods are scientifically reliable. Efforts to enhance the healthcare quality of tertiary hospitals across various cities in Guangxi should focus on the indicators of “Quality and Safety” and “Rational Medication.” Hospitals are encouraged to actively respond to the tiered diagnosis and treatment system, comply with national policies, standardize the prescription review management system, ensure the safety of clinical medication, and improve healthcare quality levels to maintain the core competitiveness of public hospitals.

## Data Availability

The raw data supporting the conclusions of this article will be made available by the authors, without undue reservation.
