# Correction: Healthcare quality evaluation of tertiary public hospitals in ethnic border regions under China's performance assessment system—based on the entropy weight TOPSIS method and RSR fuzzy set

**DOI:** 10.3389/fpubh.2025.1737625

**Published:** 2025-11-17

**Authors:** Junjie Huang, Yanlong Wu, HuiPing Pan, Haitao Yuan, Xinwen Liu, Huiyu Wang, Pingping Zeng, Zhong Tang, Pinghua Zhu

**Affiliations:** 1 Guangxi International Zhuang Medical Hospital, Nanning, China; 2 The First Affiliated Hospital of Guangxi Medical University, Nanning, China; 3 Guangxi Zhuang Autonomous Region Medical Management Service Guidance Center, Nanning, China; 4 Liuzhou Municipal Liutie Central Hospital, Liuzhou, China; 5 School of Nursing, Guangxi Medical University, Nanning, China; 6 School of Humanities and Social Sciences, Guangxi Medical University, Nanning, China

**Keywords:** tertiary public hospitals, healthcare quality, performance assessment, fuzzy set, Guangxi

Affiliation “School of Humanities and Social Sciences, Guangxi Medical University, Nanning, China” was erroneously omitted for authors Zhong Tang and Pinghua Zhu.

Authors Zhong Tang and Pinghua Zhu were erroneously assigned to affiliation “Guangxi International Zhuang Medical Hospital, Nanning, China”. This affiliation has now been removed for authors Zhong Tang and Pinghua Zhu.

The original version of this article has been updated.

